# Effects of Cognitive Knowledge and Intercultural Behavioral Skills on Cultural Stereotypes and Intercultural Affect: A Case of Elementary Students’ Perspective on Islam

**DOI:** 10.3390/ijerph182413102

**Published:** 2021-12-12

**Authors:** Gregory S. Ching, Pei-Ching Chao, Yi-Shan Kuo, Amy Roberts

**Affiliations:** 1 Graduate Institute of Educational Leadership and Development, Fu Jen Catholic University, New Taipei City 24205, Taiwan; 2 Research and Development Center for Physical Education, Health, and Information Technology, Fu Jen Catholic University, New Taipei City 24205, Taiwan; 3 Department of Education, National Chengchi University, Taipei City 116011, Taiwan; 99152513@nccu.edu.tw; 4 Guan-Pu Elementary School, Hsinchu City 30072, Taiwan; kouyishan13@gmail.com; 5 School of Teacher Education, University of Wyoming, Laramie, WY 82071, USA; aroberts@uwyo.edu

**Keywords:** Islam, Taiwan, cognitive knowledge, intercultural competence, mediation analysis, structural equation modelling

## Abstract

Two decades have passed since the September 11 attacks by Islamist militants that shocked the world. Despite this, Islamophobia remains a common phenomenon. In Taiwan, the 2014 12-year Basic Education Curriculum amendments emphasize cultural and global understanding as core competencies. With more than 6 years of implementation, it would be therefore interesting to learn what elementary school students think of Islam. Anchoring on the concepts of intercultural competency development, stereotypes are said to be related to cognitive knowledge, intercultural behavioral abilities, and attitudes. A survey instrument was developed and validated to collect information on stereotypes, skills in intercultural interaction, and attitudes toward Islam. Additionally, cognitive knowledge of Islam was also tested. A total of 712 students participated in the study. Structural equation modelling was used to test the mediating role of cognitive knowledge and intercultural behavioral skills within the relationship between cultural stereotypes and intercultural affects. Findings show that behavioral skills alone are not enough to diminish the negative aspects of stereotypes. Importantly, it is only with the help of cognitive knowledge that the relationship between stereotypes and intercultural affects are improved. It is hoped that by understanding the importance of proper curriculum content, more sustainable coexistence can be established.

## 1. Introduction

Two decades have passed since the September 11 attacks that shocked the world. Shortly after the al-Qaeda terrorists attacked the Twin Towers and the Pentagon in the United States, Muslims and Arab Americans became targets of anger and racism. Hate crimes against Muslims in the United States rose 1617 percent from 2000 to 2001 [1], marking the highest numbers of Islamophobic hate crimes on record. In the current era, Muslim communities continue to expand with a global presence in all world regions, yet discrimination against this community has not waned. The phenomenon of Islamophobia continues as a societal phobic reaction to Islam and is recognized as a global threat inflamed by fear, hatred, or prejudice against the religion of Islam and Muslim communities [2].

Although Islamophobia is present in all world regions, policies and strategies to combat it vary from country to country. The case of Taiwan, an island nation located in the Southeast Asian region, exemplifies this point. Taiwan is considered as one of the most Muslim-friendly nations outside of the Organization for Islamic Cooperation member countries [3]. Recent statistics suggest that there are currently more than 250,000 Muslims in Taiwan [4] who share a long history with everyone in Taiwan. Despite this, incidents of prejudice against Islam and the Muslim community still occur [5] side by side with regional news and media that inflame anti-Islamic sentiments [6,7,8].

While the Chinese Muslims of today have become an invisible community in Taiwan [9,10], educators and policy makers in the system have responded constructively to foster critical new understandings. More concisely, preventing and tackling Islamophobia in Taiwan is systematically embedded in the Ministry of Education elementary social studies curriculum. A primary curricular goal, as detailed in the Education for Sustainable Development for 2030 [11,12], is to increase students’ tolerance of cultural diversity. This focus was initiated in 2014 with revisions of the 12-year Basic Education Curriculum. Revisions highlighted cultural and global understanding as core competencies that Taiwanese students needed [13]. To date, the emphasis is on cultivating students’ civic responsibilities in the following areas: rule of law and human rights, international understanding, respect of cultures and ethnic differences, pursuit of social justice, global citizenship, and active participation to promote common ideals such as ecological sustainability and cultural development (p. 16) [14].

In response, this article details a descriptive survey research to measure the utility of the Taiwan Basic Education Curriculum (elementary social studies curriculum) in some of the above-mentioned areas. The overarching tenant of the study is that formal education plays an important role in student development of cognitive understandings [15] and behavioral skills [16]. The primary research goal was to examine the relationship between cultural stereotypes, cognitive knowledge, intercultural behavioral skills, and intercultural affects. Specific objectives included:

1. To validate an instrument used to measure elementary students’ cultural stereotypes, intercultural behavioral skills, and intercultural affects;
2. To determine the role of cognitive knowledge and intercultural behavioral skills in predicting intercultural affects; and
3. To determine the mediating role of cognitive knowledge and intercultural behavioral skills within the relationship between cultural stereotypes and intercultural affects.

These objectives are prompted in part by the 21st century as a time of accelerating intercultural diversity, characterized by the global movement of people, ideas, knowledge, products, images, messages, and technologies [17]. Scholars note that nationality is a major factor in developing culture, but it is only one of many considerations. The theoretical stance of social constructionists suggests that culture is learned, often subconsciously, through socialization; in addition to nationality, culture can be shaped by various gender, age, social class, occupation, and appearance stereotypes.

The Milton Bennett Developmental Model of Intercultural Sensitivity (DMIS) [18] describes standard ways in which people experience, interpret, and interact across cultures. This model is designed to place a person or group at specific stages along a continuum that indicates levels of intercultural sensitivity. Each stage is represented by varying degrees of cognitive knowledge, intercultural behavioral skills, and intercultural attitudes [19]. The six stages are indicative of particular cognitive structures with specific attitudes and skills that are associated with configurations of a worldview. The stages shift from ethnocentrism, defined as a person who uses his/her culture to experience and interpret reality, to ethno-relativism, defined as the ability to experience and interpret reality in the context of multiple cultures.

Bennett was the first in the field to argue that language training and information about specific cultures is not sufficient to prepare individuals for intercultural communication, defined as the interaction among people of different nations as well as communication between members of the same nationality but with a variety of backgrounds [20]. The way people communicate in cultural contexts often strengthens their sense of cultural identity, while cultural identity affects forms of communication and meaning. The sense of *culture* used in intercultural communication is that of *worldview* and is a generalization about how a group of people coordinate meaning and action among themselves (p. 115) [19]. An overarching goal of intercultural communication is to edify cross-cultural adaptations in formal classroom teaching and learning. Teachers and students are called to seek out and identify various cultural differences that can affect communication. By acknowledging differences, they are better able to adapt, act accordingly, and avoid misunderstandings. Attention to intercultural communication can decrease negative cultural stereotypes and develop positive communication across cultural boundaries through awareness.

That said, scholars observed that cultural sensitivity is not natural; along with this, the ability to become interculturally sensitive and competent is an active process defined as intercultural competence, meaning the capability to shift cultural perspective and appropriately adapt behavior to cultural differences and commonalities (p. 431) [21]. In essence, intercultural competence calls for depth of knowledge about and experience with a different culture in order to be more sensitive or competent in understanding of the target culture as well as other cultures in general.

In sum, this discussion informs the structure of the respective study and highlights three overarching indicators used to develop the theoretical framework:

1. Attitudes are an essential component contributing to development of knowledge and skills for inter-cultural competence. They include respect, openness, curiosity, and discovery. Openness and curiosity imply a willingness to risk beyond one’s comfort zone. Communicating respect is important to convey value toward others.
2. Knowledge can be defined as (a) cultural self-awareness of how one’s culture influences identity and personal worldview, (b) culture-specific knowledge, (c) knowledge of various world views, and (d) sociolinguistic awareness.
3. Skills, such as observation, listening, evaluating, analyzing, interpreting, and relating, are needed for the acquisition and processing of knowledge.

The summation of the attitudes, knowledge and skills, as well as the internal outcomes are demonstrated through behavior and communication, which become the visible outcomes of intercultural competence.

**Figure 1:**
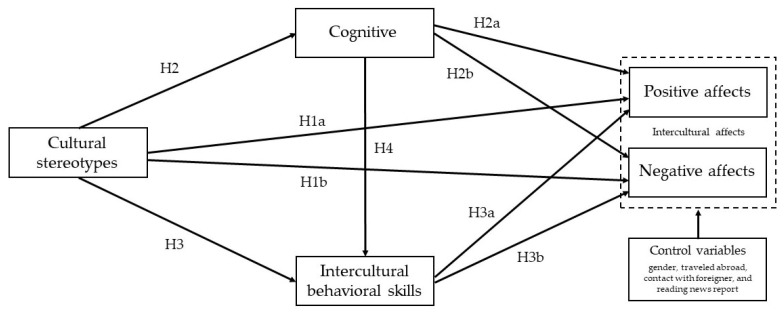
Theoretical framework of the study.

Figure 1 shows the theoretical framework based on the above discussion. Corresponding direct path relationships are denoted by H1a, H1b, H2, H2a, H2b, H3, H3a, H3b, and H4. Intercultural affects have been separated into positive and negative effects, while control variables, such as gender, travel abroad, contact with foreigners, and awareness of current events (news media), are also incorporated into the theoretical framework of the study.

Based on the theoretical framework, cultural stereotypes can be related to cognitive knowledge, behavioral abilities, and attitudes [22,23,24,25,26]. Typically, cultural stereotypes create emotional or affective prejudices based on bias [27,28]. Therefore, it can be assumed that cultural stereotypes influence how an individual treats and judges others [29]. Often, negative stereotypes result in less productive interactions with others. However, studies have shown that negative stereotypes can be minimized through the understanding and awareness of others [30,31,32]. In particular, cognitive knowledge may influence positive outcomes with cultural understanding [33,34]. In addition, cognitive knowledge and behavioral abilities or skills are important elements [20,24,35]. Empathy, listening skills, managing anxiety, and maintaining relationships are skills that help individuals interact effectively with people of varying cultural backgrounds [20] (pp. 147–165).

This framework provides a springboard for discussion. It suggests that intercultural competence is a process, meaning that there is no one point at which individuals and groups become completely interculturally competent. Intercultural competence does not *just happen*; rather, it must be intentionally addressed. In this study, intentionally addressing intercultural competence at the grade 6 level was examined through the use of the Taiwan Basic Social Studies Elementary Education Curriculum. The intent was to examine the process concerning *if* and *to what extent* the necessary knowledge, skills, and attitudes could be actualized through classroom instruction implemented with the Taiwan Basic Social Studies curriculum.

## 2. Materials and Methods

### 2.1. Study Design and Participants

The current study was designed in accordance with a descriptive approach in mind, wherein a survey was utilized for collecting the information needed to describe the phenomenon being studied [36]. In addition, the survey was designed as cross-sectional, whereby this type of data collection method is useful when examining information that cross different sections in order to infer information about the population at one given point in time [37].

The data for the current study were collected from November to December 2020 at ten public (national) elementary schools in northern Taiwan, more precisely in Hsinchu County. Hsinchu County prides itself on being the technology center in Taiwan, home to a variety of local and international companies [38]. Based on previous studies, stereotype consciousness begins at an early age of 6 years old and drastically increases till 10 years old [39]. As most Taiwanese grade 6 students are between the ages of 12 and 13, the topic of the study is in their interest area at just the right time [40]. Sampsize [41] was used to compute for the minimum sample size. According to the Taiwan Ministry of Education Statistics, there are around 197,858 grade 6 students enrolled during that school year [42]. Therefore, a minimum sample size of 384 students was required for the current study (with a 5% margin of error and 95% confidence level). The inclusion criteria called for grade 6 students who are Taiwanese citizens and are enrolled in public elementary schools.

Using the convenience sampling method, a total of 800 surveys were distributed as part of an in-class educational activity within a social studies class. In Taiwan, elementary social studies are taught to students starting from grade 3 up to grade 6. Within the 12-year Basic Education Curriculum amendments, elementary social studies emphasizes students’ development towards a sense of responsibility that recognizes diversity, values human rights, and cares about global sustainability as well as encouraging an understanding of multiple citizenships, such as ethnicity, society, locality, country, and the world [43]. Typical curriculum contents are geared towards the development of knowledge and understanding of history, religion, geography, civic, and current social issues.

As noted, the survey was administered as one component of a whole-class teaching activity. Students’ consent was voluntary; they could opt out of answering the survey questions at any time without consequence. In total, 712 volunteer grade 6 students or 89 percent of 800 responses were valid. Eighty-eight responses were removed as either invalid or as nonparticipating volunteers.

### 2.2. Measures

Participant demographics included their gender and several questions that could be answered with a simple yes or no, for example, “have you ever traveled outside the country”, “have you ever been in contact with a foreigner”, and “do you read or watch news reports”. Table 1 shows the demographic profile of the participants. The number of female participants was 349 or 49 percent, and the number of male participants was 363 or 51 percent. As summarized in Table 1, 78 percent of participants reported travel abroad experience, and 22 percent reported that they had no travel abroad experience. In close alignment, 90 percent of participants noted contact with foreigners, while 10 percent had no previous contact with foreigners. Interestingly only 35 percent of participants reported that they read or watch news reports in spare time, while 466 or 65 percent replied “no” or “none”. Access to foreign cultures and news media was noted as an important antecedent to developing intercultural competency [44,45,46].

The cognitive knowledge survey questions were organized as a checklist of fifteen Islamic culture-related facts generated from participants’ social studies class. Cronbach’s [47] alpha reliability of cognitive knowledge is computed at 0.86, implying reliable internal consistency [48]. The Islamic culture facts included items, such as “there are two major festivals in Islamic culture: Eid al-Fitr and Eid al-Adha”, “Shia and Sunni are among the main sects of Islamic culture”, and “Islamic culture requires women to wear plain burqas, scarves, or veils”.

Cultural stereotypes were defined as beliefs about a group’s characteristics and traits [49,50,51]. More specifically, the cultural stereotypes survey items pertain to the negative impressions on Islamic culture and gender issues. Using a Likert [52]-type scale, participants rated their perceived agreement with the different stereotypes using a 1 to 4 scale, wherein 1 signified strongly disagree, and 4 signified highly agree. Sample items included: “suicide bombers remind me of the Islamic culture” and “in Islamic culture, men have the right to oppress women and force them to conform to societal norms”. Cronbach’s alpha reliability of the cultural stereotypes scale was computed at 0.84, signifying satisfactory internal consistency.

Survey items pertaining to behavioral skills or abilities referred to intercultural competences, conceptualized here as what an individual can do to be effective while interacting with another culture [22,53]. Among these were various intercultural strategies and the tendencies of empathizing and being aware of cultures other than one’s own. Participants were asked to respond using a four-point Likert-type scale denoting their perceived agreement with the various behavioral tendencies. Sample items included: “I will try to learn about new and unfamiliar cultures”, “I can tell the difference between cultures”, and “when confronted with an unfamiliar culture, I feel nervous and anxious”. Cronbach’s alpha reliability of the behavioral skills scale was computed at 0.79, denoting adequate internal consistency.

Intercultural affects were defined as the various responses before, during, and after cross-cultural interactions [54]. For the current study, these included both positive and negative feelings, moods, and attitudes [22,55]. Participants were asked to respond using a four-point Likert-type scale, which indicated their level of agreement with the various positive and negative affects. Sample items included: “I am tired of learning about the Islamic culture” and “I am curious about the Islamic culture”. Cronbach’s alpha reliability of the intercultural affects scale was computed at 0.68, indicating tolerable internal consistency.

### 2.3. Data Analysis

After the survey were encoded, data missing from less than 10 percent of the total dataset were imputed using the expectation maximization algorithm [56,57]. To describe the data distribution, descriptive statistics, such as mean and standard deviation (SD), were completed. The initial exploratory factor analyses and hierarchical multiple regressions were calculated with SPSS version 20.0 (IBM, Armonk, NY, USA) on loan from the university. The intercorrelations between the variables were calculated using the Pearson correlation. Subsequent confirmatory factor analyzes and path tests were performed using Structural Equation Modeling with the aid of SPSS AMOS Version 26.0 (IBM, Armonk, NY, USA) under a Hearne Software lease agreement.

Construct validity and reliability were assessed by computing the composite reliability (CR), convergent validity (average variance extracted, AVE), discriminant validity (DV; computed by taking the square root of AVE), and heterotrait-monotrait ratio of correlations (HTMT) of the variables [58,59,60,61]. In practice, acceptable values for CR should be above 0.50 [62], while a value of more than 0.70 is much better [63]. AVE should be above 0.50 [59]; however, in cases when AVE is less than 0.50, but CR is more than 0.60, convergent validity is still acceptable [64]. DV should be greater than the variable’s interconstruct correlations [59]. HTMT should be below 0.85 for strict and 0.90 for liberal discriminant validity [65]. In determining the model fits, several criteria were used: Standardized Root Mean Square Residual (SRMR, values should be less than 0.08 to indicate a good fit); significant chi-square; chi-square divided by degrees of freedom (CMIN/df, ratio must fall between 2 and 5 to indicate a reasonable fit); Root Mean-Square Error of Approximation (RMSEA, values should be less than 0.08 to indicate a good fit), including 90 percent confidence interval (90% CI); and Goodness of Fit Index (GFI), Tucker–Lewis Index (TLI), Comparative Fit Index (CFI), all of which should have values greater than 0.90 to indicate a good fit [63,66]. Lastly, bootstrap method (sampling repeated 2000 times) was used to estimate the 95 percent CI (CI should not include zero) for significance testing of path associations and mediating effects (to determine direct and indirect effects; no mediation, partial mediation, or full mediation) [67,68].

## 3. Results

### 3.1. Validation of the Instrument

For the cultural stereotypes, a total of 12 items were initially generated. The items included various misconceptions about Islamic culture, such as those regarding negative aspects and those involving gender. Several criteria were used in the factor analysis to determine the ability of the items to be factored. First, correlations between items were examined with a correlation of not lower than 0.30 between at least one other item but that did not exceed 0.85 [69]. In the second step, two items were removed from the factor loads due to cross-loading. In practice, it is recommended that items have a primary load of at least 0.50 and no lateral load of 0.32 or more [70]. Third, the Kaiser–Meyer–Olkin (KMO) measure for the appropriateness of sampling was calculated to be 0.88, which was well above the minimum cutoff value of 0.50 [71]. Fourth, Bartlett’s sphericity test was significant with *χ*^2^ (45) = 2214.93, *p* < 0.001, signifying sampling adequacy [72]. Finally, communalities were calculated with all values above 0.40, confirming that these elements had a common variance [73].

After passing the initial check, a principal component analysis utilizing varimax rotation was then performed to identify latent variables within the remaining 10 items [74]. Results showed that the remaining 10 items loaded successfully into two variables explaining for 55.11 percent of the total variance. In addition, confirmatory factor analysis using structural equation modelling results exhibited a good model fit with SRMR = 0.035, CMIN (34) = 93.33 with *p* < 0.001, CMIN/df = 2.75, RMSEA = 0.050 (90% CI 0.038 and 0.062), GFI = 0.98, TLI = 0.97, and CFI = 0.96, wherein each of the criteria falls within the prescribed cutoff values.

In Table 2, the various cultural stereotypes variables and items are displayed together with their mean, SD, communalities, and factor loadings that are within the acceptable parameters. Two distinct variables are noted. Negative stereotype is for the undesirable cultural misconceptions towards Islam and gender stereotype is for the preconceived notion or concept about how women or men are expected to act or perform. Within the cultural stereotype variables, two items scored the highest: “among Muslim cultures, holy wars or Jihad are usually initiated for religious reasons”, with M = 2.48 (SD = 0.87), and “women are not allowed to attend school or work in Islamic culture”, with M = 2.27 (SD = 0.93).

For the intercultural behavioral skills, a total of 10 items were initially generated. The items included various intercultural strategies and the tendencies of empathizing and being aware of cultures other than their own. Following similar procedures, items were examined for their intercorrelations and their factor loadings. All of the 10 items fit well with KMO measure of sampling adequacy computed at 0.82 and Bartlett’s test of sphericity significant with *χ*^2^ (45) = 2352.29, *p* < 0.001. Communalities were also calculated with all of the values above 0.40. Principal component analysis utilizing varimax rotation showed that the 10 items loaded successfully into three variables explaining for 64.93 percent of the total variance. Lastly, confirmatory factor analysis using structural equation modelling results showed an adequate model fit with SRMR = 0.057, CMIN (32) = 172.03 with *p* < 0.001, CMIN/df = 5.38, RMSEA = 0.078 (90% CI 0.067 and 0.090), GFI = 0.95, TLI = 0.94, and CFI = 0.92, all within the acceptable ranges.

Table 3 displays the behavioral skills variables and items along with their mean, SD, communalities, and factor loadings that are within the acceptable parameters. Three distinct variables are noted. Intercultural strategies are the plans or approaches adopted to overcome cultural barriers, while intercultural awareness denotes one’s ability to comprehend both their own culture and others and especially the similarities and differences found therein. Lastly, intercultural empathy involves the ability to comprehend or share the feelings of another culture. For each of the variables, the highest item is as follows: “I will try to solve my problem with unfamiliar culture”, with M = 2.89 (SD = 0.74); “I can tell the difference between cultures”, with M = 3.11 (SD = 0.70); and “when confronted with an unfamiliar culture, I feel nervous and anxious”, with M = 2.56 (SD = 0.86).

For the intercultural affects, a total of 14 items were initially generated. These items included both positive and negative feelings, moods, attitudes, and responses based on various intercultural experiences. Following similar procedures, items were examined for their intercorrelations and their factor loadings. All of the 14 items fit well with KMO measure of sampling adequacy computed at 0.92 and Bartlett’s test of sphericity significant with *χ*^2^ (91) = 6686.73, *p* < 0.001. Communalities were also calculated with all of the values above 0.40. Principal component analysis utilizing varimax rotation showed that the 14 items loaded successfully into two variables explaining for 66.04 percent of the total variance. Lastly, confirmatory factor analysis using structural equation modelling results showed good model fit with SRMR = 0.041, CMIN (74) = 328.76 with *p* < 0.001, CMIN/df = 4.44, RMSEA = 0.070 (90% CI 0.062 and 0.077), GFI = 0.94, TLI = 0.96, and CFI = 0.95, all well within the prescribed cutoffs.

Table 4 displays the intercultural affects variables and items with their mean, SD, communalities, and factor loadings within the acceptable range. Two different variables are noted: one corresponding to the positive feelings, moods, attitudes, and responses related to various intercultural experiences and the other to the negative ones. For each of the variables, the highest item is as follows: “Islamic culture has a distinctive value”, with M = 2.86 (SD = 0.89), and “I am not interested in Islamic culture”, with M = 2.22 (SD = 0.88).

### 3.2. Correlation Analysis

Descriptive statistics and a correlation matrix for the various background demographics, cultural stereotypes, behavioral skills, and affects are presented in Table 5. In addition, each intercultural variable’s internal consistency is outlined with evidence of the validity of each, including CR, AVE, DV, and HTMT (the different cutoff criteria are described in Section 2.3).

As for the bivariate correlation analyses, results showed that, in most cases, cultural stereotypes (negative and gender) are positively correlated with negative affect while at the same time negatively correlated with positive affect. As expected, negative and gender stereotypes are positively correlated with each other. Similarly, the three intercultural behavioral skills: strategy, awareness, and empathy are also positively correlated with each other, while positive and negative affects are negatively correlated with each other. Importantly, cognitive knowledge is positively correlated with intercultural strategy, intercultural awareness, and positive affect, indicating that a greater understanding of cultural differences will lead to greater harmony between cultures. Interestingly, cognitive knowledge is also positively correlated with negative and gender stereotypes, which is quite unusual.

Moreover, reading news reports is positively correlated with negative stereotypes, gender stereotypes, and negative affect, which is quite understandable. Furthermore, reading news reports also has a positive correlation with overall intercultural strategy and awareness, which hints that both positive and negative insights on culture are provided by news sources. Additionally, results indicate that travel abroad experience and contact with foreigners are positively correlated with intercultural awareness and strategy. While, contact with foreigners is negatively correlated with negative affect, suggesting that intercultural exposure does decrease unpleasant feelings, moods, and attitudes. Lastly, gender seems to be positively correlated with negative stereotypes, travel abroad experience, and contact with foreigners, indicating that male students are more prone to having negative stereotypes.

### 3.3. Regression Analysis

Hierarchical multiple regression analyses were conducted to reveal the significant role of cognitive knowledge and intercultural behavioral skills in predicting intercultural affects (positive and negative). Variables associated with intercultural affects were entered using a three-step procedure. Firstly, to control for possible effects of background demographic, gender (0 = female, 1 = male), traveled abroad (0 = no, 1 = yes), contact with foreigners (0 = no, 1 = yes), and read news reports (0 = no, 1 = yes) were entered into the equation as control variables. In the second step, cultural stereotypes (negative and gender) were entered into the equation. Lastly, the predictor variables, cognitive knowledge, intercultural strategy, awareness, and empathy were entered into the equation.

Table 6 displays the results of the hierarchical multiple regression analyses. For the positive affect, the control variables gender (β = −0.15, *t* (707) = −4.00, *p* < 0.001) and contact with foreigners (β = 0.11, *t* (707) = 2.66, *p* < 0.01) both showed significant associations and together explained 3 percent of the variance (*F* (4, 707) = 5.54, *p* < 0.001). Negative stereotype (β = −0.13, *t* (705) = −3.05, *p* < 0.01) increased the explained variance to 4.50 percent (*F* (6, 705) = 5.45, *p* < 0.01). Lastly, the predictor variables cognitive knowledge (β = 0.20, *t* (701) = 5.55, *p* < 0.001), intercultural strategy (β = 0.47, *t* (701) = 13.05, *p* < 0.001), and intercultural empathy (β = −0.09, *t* (701) = −2.74, *p* < 0.01) all together increased the explained variance to 33.30 percent (*F* (10, 701) = 75.73, *p* < 0.001).

For the negative affect, the control variables gender (β = 0.09, *t* (707) = 2.48, *p* < 0.05), contact with foreigners (β = −0.12, *t* (707) = −3.03, *p* < 0.01), and read or watch news reports (β = 0.09, *t* (707) = 2.38, *p* < 0.05) all showed significant associations and together explained 2.70 percent of the variance (*F* (4, 707) = 4.81, *p* < 0.01), while negative stereotypes (β = 0.35, *t* (705) = 8.64, *p* < 0.001) and gender stereotypes (β = 0.13, *t* (705) = 3.42, *p* < 0.01) both increased the explained variance to 20 percent (*F* (6, 705) = 76.35, *p* < 0.001). Finally, the predictor variables cognitive knowledge (β = −0.11, *t* (701) = −2.92, *p* < 0.01), intercultural strategy (β = −0.26, *t* (701) = −6.82, *p* < 0.001), intercultural awareness (β = 0.10, *t* (701) = 2.66, *p* < 0.01), and intercultural empathy (β = 0.14, *t* (701) = 4.22, *p* < 0.01) all together increased the explained variance to 28.70 percent (*F* (10, 701) = 21.31, *p* < 0.001).

### 3.4. Mediation Analysis

**Figure 2:**
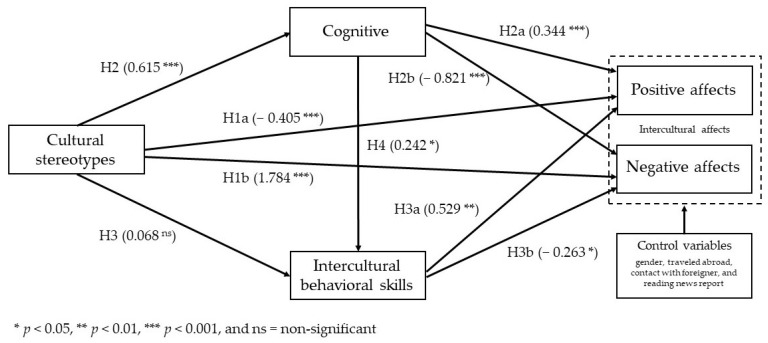
Path analytical model of the inter-relationship between the study variables.

Figure 2 shows the path analytical model tested and the associated standardized regression weights using structural equation modeling. In the figure, cultural stereotypes (negative and gender stereotypes) are regarded as the main predictor variable, while positive and negative affects are regarded as the outcome variables. Furthermore, it is also conceptualized that cognitive knowledge and intercultural behavioral skills both act as parallel and serial mediators, while gender, travel abroad experience, contact with a foreigner, and read or watch news reports were used as control variables. Structural equation modeling results exhibited a good model fit with SRMR = 0.061, CMIN (228) = 798.82 with *p* < 0.001, CMIN/df = 3.50, RMSEA = 0.059 (90% CI 0.055 and 0.064), GFI = 0.91, CFI = 0.93, and TLI = 0.91.

Table 7 and Table 8 show the direct and indirect effects of the predictor and mediators. Direct effects are denoted by H1a, H1b, H2, H2a, H2b, H3, H3a, H3b, and H4, while the indirect effects (for the parallel and serial mediations) are denoted by A1, A2, B1, B2, C, D1, D2, E1, and E2 (see Table 7 and Table 8 for more details). For the direct effects, each of the paths were all significant (except for stereotype → behavioral skills or H3, which is not supported). Cultural stereotypes were found to have significant direct negative effects on positive affects (H1a) with β = −0.405, *p* < 0.001 and significant direct positive effects on negative affect (H1b) with β = 1.784, *p* < 0.001. In other words, as cultural stereotypes often have an undesirable connotation, these results indicate that higher levels of cultural stereotypes are associated with higher levels of negative affect while at the same time associated with lower levels of positive affect. In addition, cultural stereotypes were also found to have significant direct positive effects on cognitive knowledge (H2) with β = 0.615, *p* < 0.001, while cognitive knowledge was found to have significant direct positive effects on positive affect (H2a) with β = 0.344, *p* < 0.001, at the same time exhibiting a significant direct negative effects on negative affect (H2b) with β = −0.821, *p* < 0.001. In this study, cognitive knowledge appears to produce a more favorable result, thereby improving positive affect.

Furthermore, while no significant associations were found between cultural stereotypes and intercultural behavioral skills, behavioral skills were found to have a direct positive effect on positive affects (H3a) with β = 0.529, *p* < 0.01, at the same time exhibiting a significant direct negative effect on negative affect (H3b) with β = −0.263, *p* < 0.05. Similar to cognitive knowledge, intercultural behavioral skills, which promote cross-cultural understanding, also contribute to the improvement of positive affect. Lastly, cognitive knowledge was found to have significant direct positive effects on intercultural behavioral skills (H4) with β = 0.242, *p* < 0.05.

For the total indirect effects of the parallel and serial mediations (or path analysis), all paths were significant except for B1 (stereotype → behavioral skills → positive affects) and B2 (stereotype → behavioral skills → negative affects), in which intercultural behavioral skills was used as mediator. Importantly, path C (stereotype → cognitive → behavioral skills) exhibited full mediation with total effects of β = 0.149, *p* < 0.01, while the rest were partial mediations. In other words, since the direct effect between cultural stereotypes and intercultural behavioral skills was not supported, cognitive knowledge fully mediates the relationship between cultural stereotypes and intercultural behavioral skills.

For the serial mediations, both paths E1 (stereotype → cognitive → behavioral skills → positive affects) and E2 (stereotype → cognitive → behavioral skills → negative affects) were significant with β = 0.149, *p* < 0.01, respectively, indicating that cognitive knowledge and intercultural behavioral skills mediated the relationship between cultural stereotypes and intercultural affects in a sequential manner.

## 4. Discussion

### 4.1. Mean Scores of Variables and Their Intercorrelations

The current study validated several variables used to describe and measure the Taiwanese elementary students’ cultural stereotypes, intercultural behavioral skills, and intercultural affects. It was found that the proposed variables negative stereotype, gender stereotype, intercultural strategy, intercultural awareness, intercultural empathy, negative affect, and positive affect are all psychometrically sound. Furthermore, the results of this study are actually somewhat encouraging, with an overall Islam-centric cultural stereotype computed at 2.18, which indicates a general disagreement regarding the various stereotype items. Meanwhile, the average negative affects score is 2.06, indicating a lower likelihood of manifestations of undesirable intercultural attitudes and interactions. In reality, the low values demonstrate rather positive student attitudes toward cultural stereotypes. These findings might be relative to the influx of the so-called new migrant partners (or foreign husbands or wives) for the past several decades, whose presence is fast becoming a norm for multiculturalism and also a source of cultural resilience [75,76]. Additionally, as the study participants reside in an area where foreign individuals are readily available [13], the perceived worldview of these individuals changes in accordance with Milton’s model (DMIS).

In addition, overall intercultural behavioral skills are computed at 2.73, which indicates students are relatively inclined towards effective intercultural strategies. In a similar way, average positive affects is calculated at 2.55, showing similar positive attitudes and vibes towards intercultural exchanges. As for the cognitive knowledge, most students can recollect about six out of the 15 facts about Islam that were included within their social studies classes. The top three cognitive knowledge items students are familiar with are “in Islamic culture, eating pork and animal blood is prohibited”, “women have to wear plain burqas, scarves, and veils in Islamic culture”, and “Islam is a religion of Islam, Muslim, or Halal”, while the least understood facts about Islamic culture is that “Shias and Sunnis are two major sects within Islam”.

Regarding the intercorrelations, aside from the anticipated positive correlations within the negative-inclined variables (negative and gender stereotypes, and negative affect) and among the positive-associated measures (intercultural strategy, awareness, and empathy, and positive affect) and negative correlations between positive and negative affects, several interesting findings are noted. Findings show that cognitive knowledge correlates positively with intercultural strategy and awareness as well as positive affect, but it also positively correlated with gender and negative stereotypes, which is rather unusual. According to this finding, cognitive knowledge has implications for both positive and negative aspects of intercultural understanding. Intercultural studies have previously found that increased knowledge of another group correlates with increased liking for that group and, by extension, a reduction in stereotypes and prejudice [77]. However, further studies on intercultural contact theories have proven that these conditions do not essentially diminish stereotypes or prejudice towards others [78,79] and might be dependent on prior contact experience or social ties between foreigners [80]. This may also explain why intercultural awareness and empathy are positively correlated with negative and gender stereotyping.

The correlations for travel abroad experience, contact with foreigners, and read or watch news reports with the other variables are also worthy of discussion. Findings showed that travel abroad experience and contact with foreigners are positively correlated with each other while at the same time positively correlated with intercultural strategy and awareness. These findings are consistent with previous research regarding instances of tourism or study abroad experiences that resulted in a greater intercultural understanding [81,82,83]. Moreover, reading or watching news reports seems to be positively correlated with negative affect, gender, and negative stereotypes, providing support to past studies demonstrating news media’s detrimental impact on Islam [6,8]. The study did, however, find that reading the news is positively correlated with intercultural strategy and awareness, which suggests that not all news media have an adverse effect on intercultural interactions. It is possible that some Taiwanese view media as a way to relax rather than as a source of information [84]. The fact remains that as the participants are exposed to cultural differences through actual out-class experiences (exposure to news media, travel abroad, and/or contact with foreigners) and in-class cognitive knowledge, the participants’ understanding of the cultural context gradually expands and grows.

### 4.2. Regressions

To determine the role of cognitive knowledge and intercultural behavioral skills in predicting intercultural (positive and negative) affect, hierarchical multiple regressions were computed. To add, several components were used as controlling variables, such as gender, travel abroad experience, contact with foreigners, and read or watch news reports and together with negative and gender stereotypes. These necessary steps were taken in order to remove their effects on the prediction of positive and negative effects [85,86]. Table 9 presents the different predictors of intercultural affect, along with their significance and direction of association with the dependent variable.

Results show positive affect will most likely associated with female students, contact with foreigners, less negative stereotypes, increased cognitive knowledge and intercultural strategies while accompanied by less intercultural empathy. In terms of the negative effects, these are mostly associated with male students, less contact with foreigners, and a higher tendency toward reading or watching news reports. Furthermore, negative affect is associated with higher negative and gender stereotyping with lesser cognitive understanding and usage of intercultural strategies while at the same time possessing greater intercultural awareness.

After discounting the effects of the control variables and cultural stereotypes, careful comparison and analyses of the results in some ways confirms what Deardorf [25] and Bennett [22] proposed: that positive affect is most likely associated with increased cognitive knowledge and understanding of the foreign culture. Likewise, negative affect is linked with less cognitive knowledge and misunderstanding of the foreign culture. Of particular interest is the role played by intercultural empathy. For the current study, intercultural empathy was defined as an ability to understand and share feelings from another culture. Researchers have found that empathy can have a significant impact on attitudes [87,88]; however, despite its ability to produce positive affect, studies have also found that empathy cannot prevent stereotypes and prejudice [89,90]. Hence, too much intercultural empathy can, therefore, lead to negative effects. Furthermore, the findings also revealed that intercultural awareness is associated with negative effects. In contrast to the current findings, previous studies have found that intercultural awareness could promote a better understanding of others, resulting in positive effects [91,92]. However, it could appear that within the current study, intercultural awareness might not directly cause negativity toward others but instead increase the focus and preference towards one’s own culture [93,94]. The DMIS argued that information about a particular culture is not sufficient to prepare individuals for intercultural communication. Cultural identity is only created through contextualizing experiences and reflecting on them. Thus, to the extent that the students are exposed to news media, careful contextualization is required, and more importantly, a non-biased discussion led by the teacher in conjunction with cognitive knowledge (related to classroom lessons) should be appropriate to create a deeper understanding of intercultural differences.

### 4.3. Mediation Effects and Structural Path

Structural equation modelling was used to determine the mediating role of cognitive knowledge and intercultural behavioral skills within the relationship between cultural stereotypes and intercultural affects. Specifically, associations between the variables including path analyses (parallel and serial) mediations were tested towards predicting the outcome variables (positive and negative affects simultaneously). Results indicate that when considering a direct approach (stereotype → positive affect and stereotype → negative affect), an increased tendency for cultural stereotypes (which are supposed to be mostly negative) leads to negative affect; alternatively, a lack of stereotypes leads to positive affect. These findings are consistent with the majority of studies that show cultural stereotypes directly impact individuals’ attitudes [95,96,97,98,99].

For the mediating role of cognitive knowledge, findings show positive affects actually increase as a result of cognitive knowledge while negative affects decrease. These findings emphasize the importance of an educational approach in promoting intercultural understanding. In Taiwan, previous research has noted the importance of lesson activities that build intercultural understanding [100] and the importance of a culturally accepting school environment that also contributes positively to developing intercultural competence [101,102]. As for the serial mediation, since cultural stereotypes and intercultural behavior skills are not directly associated (as seen in Table 7), the only viable approach is to link cognitive knowledge and intercultural behavior skills sequentially. Wherein, positive affect and negative affect are both impacted in a good way. Based on the current findings, it has been revealed that cultural perspectives can be shifted with the help of cognitive knowledge and intercultural skill sets. Within the classroom, it is common for students to approach global issues from a local perspective. Consequently, it is the teacher’s responsibility to encourage students to approach the same issue from a variety of cultural perspectives by asking questions and inviting students to express both their opinions and differences in class. By doing so, students are able to connect the cognitive knowledge that they have acquired previously with the appropriate intercultural behavior.

## 5. Conclusions

This study contributes to the body of intercultural research and discourses addressing the relationship between cultural stereotypes, cognitive knowledge, intercultural behavioral skills, and intercultural affects. Implications suggest that formal education plays an important role in student development of cognitive understandings [15] and behavioral skills [16]. Global implications suggest that behaviors must be adapted to each new specific curricular encounter with culture. Knowing that intercultural communication can occur between members of the same nationality, educators and students encounter intercultural communication daily, even when they are within homogeneous classroom settings.

Findings suggest that Taiwanese grade 6 students are moderately familiar with the necessary knowledge, skills, and attitudes needed to avoid negative cultural stereotyping of the Muslim community in Taiwan. Implications support the timeless notion that participants can better communicate when they are able to understand the intentions of one another in non-evaluative or stereotypical ways. Suggesting that intercultural behavioral skills alone are not sufficient to diminish the negative aspects of stereotypes; it is only with the addition of cognitive knowledge that intercultural affects are benefited. An important consideration for teachers and caregivers is that cognitive knowledge and the tendency for students to read or watch news reports have both positive and negative connotations in the intercultural sphere.

Many scholars agree that effective intercultural understanding in school settings is affected by the design of the curriculum and the inclusion of current events as well as the appropriate participation and explanation of teachers. At first glance, it appears logical to recommend that teachers continue to rely on the Taiwan Basic Social Studies Education Curriculum as an overarching resource. However, this might not be feasible for teachers who want to create neutral platforms for intercultural discussions and understanding. The Taiwan Basic Social Studies Education Curriculum is tailored to the *local-national nexus*, but the basic contours of change are rooted in broader global patterns, meaning that the pedagogical stance of teachers should extend beyond a national enterprise. It is evident that the local and national values are becoming an increasingly important issue of discussion even within the elementary schools. In the case of Taiwan, the national elements embedded in the Basic Social Studies Education Curriculum should not disappear, but perhaps attention to knowledge, in terms of students reading and watching news reports, should be considered as an opportunity to expand the intercultural sphere of diversity and culture.

The promise of mentoring young Taiwan students to become interculturally competent is hinged on two provisions. Firstly, teachers require ongoing professional development of research-based pedagogies. The grade 6 students relied on teacher-directed textbook learning. Mandating pedagogies that are shaped by methods to actively engage students with key concepts, skills, and foundations of intercultural communication are important but will mean very little if teachers are not trained in the respective areas. Secondly, Taiwan teachers should have active roles with curricular efforts currently in progress. A recommendation is to encourage a bottom-up curriculum strategy. The bottom-up strategy provides a common ground for teachers to reflect on their practices, to experiment with innovation, and to speculate on ideas as theories of teaching. Teachers take on the role of curriculum developers rather than curriculum users. While the first position speaks to issues of empowerment and social justice, the latter suggests issues of control and dependency. The focus of control over what counts as valid educational knowledge is shifted from external agencies to the schools and teachers’ classrooms. This shift could prompt teachers to search for new ways to develop intercultural competence in their classrooms.

With this, it is important to mention limitations of the study. The research was limited in noting that only grade 6 students in public elementary schools in northern Taiwan were included. A future study could compare grade levels across all regions of Taiwan. Moreover, the survey data were collected as part of an educational activity; hence, some students may not have taken it seriously. With that being said, more research is needed to examine how teachers navigate cultural and political structures through their pedagogy and across national contexts. In addition, information with regards to the impact of singular student background, the family’s student environment, and the impact of the elementary schools teaching staff and school culture can also be a point of future discussion, hence giving grounds for a strong rationale for follow up perhaps by way of detailed interview studies with both teachers and students.

In conclusion, this study offers a reference for understanding how students and teachers in the Taiwan system engage with specific topics toward becoming interculturally competent. Overarching takeaways include understanding that while there are many different roads to the same ends, all should be considered by the extent to which they demonstrate potential for students and teachers.

## Figures and Tables

**Table 1:** Demographic profile of the participants.

Demographics	Classification	*n*	%
Gender	Female	349	49
	Male	363	51
Travel abroad experience	Yes	558	78
	No	154	22
Contact with foreigners	Yes	642	90
	No	70	10
Read or watch news reports	Yes	246	35
	No	466	65

Note. *N* = 712.

**Table 2:** Item means, communalities, and factor loadings for cultural stereotypes.

Variables and Items (Variance Explained)	Mean	SD	Communalities	FL
Negative stereotype (27.69%)	2.24	0.62		
I believe that terrorist attacks are linked to Islamic culture	2.32	0.83	0.69	0.81
Suicide bombers remind me of the Islamic culture	2.08	0.82	0.65	0.78
War continues in the Middle East due to the various Islamic faiths that exist in the region	2.20	0.84	0.56	0.73
Among Muslim cultures, holy wars or Jihad are usually initiated for religious reasons	2.48	0.87	0.45	0.62
I consider most Islamic nations to be backwards and poor	2.11	0.80	0.42	0.62
Gender stereotype (27.41%)	2.27	0.93	0.44	0.62
I believe that the veil is a symbol of feminist backwardness for women in the Islamic culture	2.06	0.98	0.66	0.80
In Islamic culture, men have the right to oppress women and force them to conform to societal norms	2.18	0.99	0.58	0.75
Muslim men are allowed to have up to four wives, which leads to a low status for women	2.09	0.90	0.60	0.73
Women who commit wrongdoing are sometimes sentenced to death within the Islamic culture to preserve their honor	1.95	0.93	0.46	0.65
Women are not allowed to attend school or work in Islamic culture	2.27	0.93	0.44	0.62
Overall cultural stereotypes	2.18	0.57		

Notes. *N* = 712. SD, standard deviation; FL, factor loading. Extraction method: principal component analysis. Rotation method: Varimax with Kaiser normalization. Rotation converged in 3 iterations. Overall Cronbach’s alpha reliability for cultural stereotypes = 0.84.

**Table 3:** Item means, communalities, and factor loadings for intercultural behavioral skills.

Variables and Items (Variance Explained)	Mean	SD	Communalities	FL
Intercultural strategy (31.50%)	2.75	0.63		
I will try to adapt to new cultures	2.66	0.87	0.59	0.76
I will try to learn about new and unfamiliar cultures	2.71	0.84	0.55	0.74
When living in another culture, I am willing to adjust	2.75	0.83	0.69	0.82
I will try to solve my problem with unfamiliar culture	2.89	0.74	0.52	0.59
I will adapt my behavior pattern to suit different cultures	2.75	0.82	0.64	0.79
Intercultural awareness (18.30%)	2.94	0.60		
I can tell the difference between cultures	3.11	0.70	0.73	0.76
I am able to see the differences between my culture and others’	3.01	0.73	0.73	0.73
I am aware of the cultures that I have difficulty adapting to	2.71	0.90	0.57	0.67
Intercultural empathy (15.12%)	2.49	0.73		
When confronted with an unfamiliar culture, I feel nervous and anxious	2.56	0.86	0.75	0.85
When possible, I will avoid unfamiliar cultural situations	2.42	0.86	0.72	0.82
Overall intercultural behavioral skills	2.73	0.47		

Notes. *N* = 712. SD, standard deviation; FL, factor loading. Extraction method: principal component analysis. Rotation method: Varimax with Kaiser normalization. Rotation converged in 5 iterations. Overall Cronbach’s alpha reliability for intercultural behavioral skills = 0.79.

**Table 4:** Item means, communalities, and factor loadings for intercultural affects.

Variables and Items (Variance Explained)	Mean	SD	Communalities	FL
Negative affects (28.50%)	2.06	0.68		
I detest Islamic culture	2.05	0.83	0.75	0.83
I am tired of learning about the Islamic culture	1.97	0.78	0.78	0.86
I believe Islamic culture is worthless	1.81	0.77	0.66	0.79
I don’t associate myself with the Islamic culture	2.10	0.89	0.75	0.84
I am not interested in Islamic culture	2.22	0.88	0.46	0.68
I do not want to be exposed to Islamic culture	2.21	0.91	0.54	0.70
Positive affects (37.54%)	2.55	0.72		
I am interested in learning more about Islamic culture	2.57	0.91	0.67	0.78
I am curious about the Islamic culture	2.46	0.89	0.70	0.80
I will pay attention to activities relating to Islamic culture	2.37	0.85	0.67	0.80
I would like to learn more about the Islamic culture from my teacher	2.64	0.90	0.68	0.81
I am interested in things related to Islamic culture	2.33	0.83	0.73	0.83
I am happy to help others learn more about the Islamic culture	2.49	0.88	0.73	0.85
Islamic culture has a distinctive value	2.86	0.89	0.61	0.76
I believe that Islamic culture is about pursuing peace	2.70	0.94	0.51	0.69
Overall intercultural affects	2.31	0.37		

Notes. *N* = 712. SD, standard deviation; FL, factor loading. Extraction method: principal component analysis. Rotation method: Varimax with Kaiser normalization. Rotation converged in 3 iterations. Overall Cronbach’s alpha reliability for intercultural affects = 0.68.

**Table 5:** Descriptive statistics, intercorrelations, reliabilities, and validities for the study variables.

Variables	Mean	SD	1	2	3	4	5	6	7	8	9	10	11	12
1. Negative stereotype	2.24	0.62	0.79	0.510 **	ns	0.142 **	0.184 **	−0.130 **	0.418 **	0.247 **	0.231 **	ns	ns	0.086 *
2. Gender stereotype	2.11	0.70		0.79	ns	0.097 **	0.142 **	ns	0.313 **	0.178 **	0.143 **	ns	ns	ns
3. Strategy	2.75	0.63			0.83	0.482 **	0.111 **	0.500 **	−0.209 **	0.267 **	0.133 **	0.100 **	0.185 **	ns
4. Awareness	2.94	0.60				0.65	0.273 **	0.233 **	ns	0.231 **	0.113 **	0.106 **	0.233 **	ns
5. Empathy	2.49	0.73					0.62	ns	0.221 **	ns	ns	ns	ns	ns
6. Positive affects	2.55	0.72						0.93	−0.445 **	0.251 **	ns	ns	ns	−0.142 **
7. Negative affects	2.06	0.68							0.89	ns	0.085 *	ns	−0.090 *	0.085 *
8. Cognitive	6.31	4.04								0.86	0.361 **	ns	0.136 **	ns
9. News ^1^	0.35	0.48										0.102 **	0.101 **	ns
10. Travel ^1^	0.78	0.41											0.342 **	0.133 **
11. Contact ^1^	0.90	0.30												0.139 **
12. Gender ^2^	0.51	0.50												
Minimum value			1	1	1	1	1	1	1	1	0	0	0	0
Maximum value			4	4	4	4	4	4	4	15	1	1	1	1
Composite reliability (CR)			0.80	0.79	0.83	0.73	0.72	0.93	0.89					
Average variance extracted (AVE)			0.45	0.44	0.50	0.50	0.59	0.61	0.58					
Discriminant validity (DV) ^3^			0.67	0.66	0.70	0.71	0.77	0.78	0.76					
Heterotrait-monotrait ratio of correlations (HTMT)			0.644		0.157 to 0.677			0.491						

Notes. *N* = 712. Numbers 1 to 12 correspond to the variables. SD, standard deviation. Overall Cronbach’s alpha reliability of the entire survey = 0.86. ^1^ Variable is coded as binary: 0, no; 1, yes. ^2^ Variable is coded as binary: 0, female; 1, male. ^3^ DV is computed using the squared root of AVE. Pearson correlation coefficients are above the diagonals with ns = non-significant, * *p* < 0.05, and ** *p* < 0.01. Internal consistency values: Cronbach’s alpha coefficients are on diagonals.

**Table 6:** Hierarchical multiple regression analyses for predicting intercultural affects.

	Predictors	*F* Change	*t*	df	B	SE	β	VIF	R^2^ Change
A. Dependent variable: Positive affects									
I.	Constant				2.50	0.09			0.030
	Control variables	5.54 ***		4707					
	Gender		−4.00 ***		−0.22	0.05	−0.15	1.03	
	Travel		−1.32		−0.09	0.07	−0.05	1.15	
	Contact		2.66 **		0.25	0.10	0.11	1.15	
	News		0.29		0.02	0.06	0.01	1.02	
II.	Cultural stereotypes	5.45 **		6705					0.015
	Negative stereotype		−3.05 **		−0.16	0.05	−0.13	1.41	
	Gender stereotype		0.41		0.02	0.04	0.02	1.36	
III.	Predictors	75.73 ***		10,701					0.288
	Cognitive knowledge		5.55 ***		0.04	0.01	0.20	1.30	
	Intercultural strategy		13.05 ***		0.53	0.04	0.47	1.37	
	Intercultural awareness		0.53		0.02	0.05	0.02	1.47	
	Intercultural empathy		−2.74 **		−0.09	0.03	−0.09	1.14	
B. Dependent variable: Negative affects									
I.	Constant				2.17	0.09			0.027
	Control variables	4.81 **		4707					
	Gender		2.48 *		0.13	0.05	0.09	1.03	
	Travel		0.59		0.04	0.07	0.02	1.15	
	Contact		−3.03 **		−0.27	0.09	−0.12	1.15	
	News		2.38 *		0.13	0.05	0.09	1.02	
II.	Cultural stereotypes	76.35 ***		6705					0.173
	Negative stereotype		8.64 ***		0.38	0.04	0.35	1.41	
	Gender stereotype		3.42 **		0.13	0.04	0.13	1.36	
III.	Predictors	21.31 ***		10,701					0.087
	Cognitive knowledge		−2.92 **		−0.02	0.01	−0.11	1.30	
	Intercultural strategy		−6.82 ***		−0.27	0.04	−0.26	1.37	
	Intercultural awareness		2.66 **		0.12	0.04	0.10	1.47	
	Intercultural empathy		4.22 ***		0.13	0.03	0.14	1.14	

Notes. *N* = 712, *t*, for within-set predictors; df, degrees of freedom; B, unstandardized coefficients; SE, standard error; β, standardized coefficients; VIF, variance inflation factor (values should be < 10). Gender was coded as binary, with 0, female; 1, male. Travel, contact, and news were coded in binary, with 0, no; 1, yes. * *p* < 0.05, ** *p* < 0.01, and *** *p* < 0.001.

**Table 7:** Direct effects of the predictor variables and mediators.

Paths	Direct Effects	B	SE	β	95% CI	*p*
H1a	stereotype → positive affects	−1.315	0.195	−0.405	(−0.552, −0.256)	<0.001
H1b	stereotype → negative affects	5.618	1.155	1.784	(1.333, 2.598)	<0.001
H2	stereotype → cognitive	11.130	1.704	0.615	(0.523, 0.709)	<0.001
H2a	cognitive → positive affects	0.062	0.010	0.344	(0.250, 0.441)	<0.001
H2b	cognitive → negative affects	−0.143	0.030	−0.821	(−1.381, −0.536)	<0.001
H3	stereotype → behavioral skills	0.027	0.035	0.068	(−0.110, 0.276)	0.547
H3a	behavioral skills → positive affects	4.347	1.446	0.529	(0.432, 0.625)	0.001
H3b	behavioral skills → negative affects	−2.103	0.930	−0.263	(−0.496, −0.094)	0.019
H4	cognitive → behavioral skills	0.005	0.002	0.242	(0.111, 0.373)	0.015

Notes. B, regression coefficient; SE, standard error; β, standardized coefficient; CI, confidence interval.

**Table 8:** Indirect effects of the predictor variables and mediators.

Paths	Indirect Effects	B	SE	β	95% CI	*p*
	Parallel mediations					
A1	stereotype → cognitive → positive affects	0.685	0.097	0.211	(0.447, 1.041)	0.001
A2	stereotype → cognitive → negative affects	−1.590	0.076	−0.505	(−3.61, −0.811)	0.001
B1	stereotype → behavioral skills → positive affects	0.117	0.066	0.036	(−0.168, 0.56)	0.550
B2	stereotype → behavioral skills → negative affects	−0.056	0.259	−0.018	(−0.435, 0.034)	0.490
C ^1^	stereotype → cognitive → behavioral skills	0.059	0.053	0.149	(0.017, 0.138)	0.005
D1	cognitive → behavioral skills → positive affects	0.023	0.044	0.128	(0.011, 0.037)	0.004
D2	cognitive → behavioral skills → negative affects	−0.011	0.023	−0.064	(−0.02, −0.007)	0.003
	Serial mediations					
E1	stereotype → cognitive → behavioral skills → positive affects	0.255	0.163	0.149	(0.133, 0.468)	0.003
E2	stereotype → cognitive → behavioral skills → negative affects	−0.123	0.335	0.149	(−0.258, −0.067)	0.005

Notes. ^1^ Full-mediation. B, regression coefficient; SE, standard error; β, standardized coefficient; CI, confidence interval.

**Table 9:** Summary of results for the hierarchical multiple regressions.

Predictors	Positive Affect	Negative Affect
Control variables		
gender	✓(−)	✓(+)
travel abroad experience		
contact with foreigners	✓(+)	✓(−)
read or watch news reports		✓(+)
Cultural stereotypes		
negative stereotype	✓(−)	✓(+)
gender stereotype		✓(+)
Main predictors		
cognitive knowledge	✓(+)	✓(−)
intercultural strategy	✓(+)	✓(−)
intercultural awareness		✓(+)
intercultural empathy	✓(−)	✓(+)

Notes. ✓ = significant predictors. (−) negative or (+) positive association with the dependent variable.

## Data Availability

Data for the current study are available at https://doi.org/10.6084/m9.figshare.16632697.v1 (accessed on 17 September 2021).
